# Effects of Acupoint Application Therapy with TianGui Powder on Osteoporosis in Ovariectomized Rats through TGF‐β1 and Smad2/3 Signaling Pathway

**DOI:** 10.1111/os.12427

**Published:** 2019-03-04

**Authors:** Xiao‐Sheng Lin, Hai‐yan Wang, Zhen Zhang, Han‐Jiao Liu, Zhen Qu, Ke‐Liang Wu, Qing‐Hua Xiao, Jian‐Zong Zhu, Ping Zhang

**Affiliations:** ^1^ ShenZhen Bao’An Shajing People's Hospital Guangzhou Medical University Shenzhen China; ^2^ ShenZhen Bao’an Traditional Chinese Medicine Hospital Shenzhen China; ^3^ Postdoctoral Station in Dongguan & Guangzhou University of Chinese Medicine Cooperative Academy of Mathematical Engineering for Chinese Medicine Dongguan China; ^4^ Guangzhou University of Chinese Medicine School of Shenzhen Bao’An Shajing People's Hospital Guangzhou China; ^5^ Department of Anatomy and Histology, School of Basic Medical Sciences Tianjin Medical University Tianjin China

**Keywords:** Bone metabolism, Postmenopausal osteoporosis, Smad‐2/3 signaling pathway, TGF‐β1, TianGui Powder, Traditional Chinese medicine

## Abstract

**Objectives:**

To explore the effects of acupoint application therapy (AAT) with TianGui Powder (TGP) on the expressions of the transforming growth factor β1 (TGF‐β1) and Smad‐2/3 signaling pathway in ovariectomized osteoporosis rats.

**Methods:**

Sixty rats were randomly divided into four groups: normal group (group A), model group (group B), TGP group (group C), and Western medicine group (group D). Group A had only the corresponding amount of adipose tissue around the ovary removed; rats in the other groups had bilateral ovariectomies. After 1 week, groups A and B were given 1 mL/100 mg normal saline solution by gavage, group C was treated with AAT with TGP on ShenQue acupoint (0.2 piece/rat, 6 h/time, 1 time/d) and group D was given calcium carbonate vitamin D3 (36 mg/kg/d) and alfacalcidol (0.05 μg/kg/d) tablet suspension. In this study, the bone mineral density (BMD) , the levels of BALP, TRAP‐5b, and BGP in serum and the changes in bone histomorphology was detected. For acquiring lumbar experimental data, the expression of TGF‐β1, Smad‐2/3 proteins and mRNA of TGF‐β1and Smad‐2/3 were assessed. After 12 weeks, the data were collected for analysis.

**Results:**

Compared with group A, the bone trabecula was thinner and significantly reduced in other groups. The result of BMD improved significantly in both groups C and D compared to group B after intervention (*P* < 0.05). In contrast, compared to group B, the levels of BALP, TRAP‐5b, and BGP significantly declined in both groups C and D. In group C, the results of protein expressions in TGF‐β1, Smad‐2/3 were 2.870 ± 0.270, 1.552 ± 0.111, and 1.420 ± 0.079, respectively. In groups C and D, those indications significantly declined compared to group B (*P* < 0.01). In group C, the level of mRNA expressions of TGF‐β1, Smad‐2/3 were 1.872 ± 0.177, 1.672 ± 0.086, and 1.790 ± 0.136, respectively. Compared with group B, those indications had significant difference in groups C and D (*P* < 0.05).

**Conclusion:**

Acupoint application therapy with TGP could significantly improve the BMD. The TGF‐β1 and Smad‐2/3 signaling pathway could be a therapeutic target of TGP in postmenopausal osteoporosis rats.

Postmenopausal osteoporosis (PMOP) is an estrogen deficiency‐induced metabolic bone disorder disease in menopausal women, which is characterized by low bone mass, bone microstructure damage, and an increased risk of fracture^1^. Estrogen deficiency may break up the balance between bone formation and resorption, and it appears to be a main cause of osteoporosis in women after the fifth decade. PMOP may increase the risk of fractures and reduce quality of life, which would cause considerable burden on patients and the health‐care system^2^. It is one of the leading causes of disability and death in elderly patients and has become a serious public health problem^3^. Melton *et al*. show that the risk of women suffering from osteoporotic fracture (40%) is higher than the total risk of breast cancer, endometrial cancer, and ovarian cancer^4^. Hernlund *et al*. report that in 2010 more than 27.5 million people had osteoporosis; moreover, in excess of 3.5 million people suffered from fragility fractures secondary to osteoporosis among the 27 countries in the European Union^5^.

The drugs for treatment of osteoporosis include bisphosphonates, parathyroid hormones, and hormone replacement therapy; however, the side effects of these drugs could increase the risk of cancer, stroke, and heart attack^6^, ^7^, ^8^. Thus, adopting a new treatment to reduce fracture risks for this disease is vitally important.

Traditional Chinese medicine (TCM) has been used in various diseases for thousands of years, with positive therapeutic effects and few side effects in China. According to TCM theory, the kidney governs bones and plays a leading role in bone growth, development, and formation. Recently, the application of TCM for the treatment of osteoporosis has attracted increasing attention.

Acupoint application therapy (AAT) is one of the means of treatment in TCM. This treatment attaches the drugs on the acupoints, and the body can absorb it through skin. On its own or combined with Western medicine, it has superior efficacy and fewer side effects than Western medicine alone. Many studies^9^, ^10^, ^11^, ^12^ have indicated that AAT can regulate bone metabolism endocrine, improve bone metabolism, and increase bone density. One such treatment is AAT combined with TianGui Powder (TGP), an integral part of traditional Chinese medicine, which has achieved satisfactory clinical outcomes. Previous studies have confirmed that TGP applied on the umbilical region can effectively improve BMD, blood TGF‐β1 content, and clinical symptoms in perimenopausal female patients^13^, ^14^. It was thought that TGF‐β and Smads proteins regulatory factors can increase the osteoblast activity and play an important role in bone remodeling^15^. However, these AAT with TGP trials provide insufficient evidence to determine the effectiveness of rebuilding osteoporotic bone because of failure to report important outcomes in signaling pathway mechanisms.

The main objectives of the present study were: (i) to observe the morphology of bone tissue under electron microscope after comprehensive intervention; (ii) to compare the BMD and bone metabolism markers among four groups; and (iii) to explore the effect of AAT with TGP on the expressions of the TGF‐β1 and Smad‐2/3 signaling pathway in ovariectomized osteoporosis Rats.

## Materials and Method

### *Animal Models*

A total of 60 female Sprague–Dawley rats (420 ± 30 g, 6 months old) were provided by the Experimental Animal Center of HuBei TCM, Wuhan, China. All rats were housed in ventilated cages (4 rats/cage) with free access to pellet feed and water under 12 h light/dark cycle, 24 ± 2°C laboratory temperature, and 58% ± 4% relative humidity.

All 60 rats were randomly assigned to 4 groups with 15 rats each: a normal group (Group A), a model group (Group B), a TGP group (Group C), and a Western medicine group (Group D). All rats were given 0.2 mL/100 g i.p. anesthesia injection with 3% pentobarbital sodium solution. Rats were given sham surgery with abdominal incision with the corresponding quantity of adipose tissue around the ovary being removed in group A, while other groups were treated with abdominal incision and bilateral ovariectomy. Penicillin was given by 80 u/d i.m. injection during the 3 days after the operation in case of infection. TGP (supplied by Shajing People's Hospital of Bao’an Shenzhen) includes Colla cornus cervi (Lujiaojiao), Cistanche (Roucongrong), *Achyranthes bidentate* (Huainiuxi), and *Eucommia ulmoides* (Duzhong). Group C was treated by AAT with TGP on the umbilical region (6 h/time, 1/d). In group D, all rats were fed calcium carbonate vitamin D3 (36 mg/kg/d) and alfacalcidol (0.05 μg/kg/d) by tablet suspension (Wyeth Pharmaceutical H10950029). Groups A and B were given 1 mL/100 mg normal saline solution.

One month after the establishment of the model, the two medication groups were treated with corresponding medicines. After 12 weeks, all rats were anaesthetized with 3% pentobarbital solution (0.2 mL/100 g) for blood collection, and then killed by bloodletting; we extracted the centrums and bilateral femora for further research.

### *Serum of Bone Metabolism Markers*

Levels of BALP, TRACP‐5b, and BGP in the serum were measured following the instructions in the ELISA kits (Wuhan Cusabio Biotech, China). All bone metabolism markers were assessed at an absorbance value of 450 nm with an enzyme‐linked immune detector (EnSpire, Perkin Elmer Instrument, USA).

### *Bone Mineral Density and Histopathological Changes*

Bone mineral density (g/cm^2^) of L1‐3 bone was measured by dual energy X‐ray absorptiometry. Moreover, the left distal femurs were decalcified by ethylenediamine tetraacetic acid solution (EDTA) with 10% formalin for 4 weeks and then embedded in paraffin. The paraffin‐embedded femur tissues were sectioned into 4‐μm‐thick specimens for hematoxylin and eosin (HE). The stained femurs were observed and imaged with the light microscope (Nikon Eclipse80i, Tokyo, Japan).

### *Electron Microscope Scanning*

In addition, the right distal femurs were decalcified by EDTA with 10% formalin for 4 weeks and then embedded in paraffin. The paraffin‐embedded femur tissues were sectioned into 4‐μm‐thick specimens for electron microscope scanning (model JSM‐6510LV, from JEOL).

### *Detection on the Protein Expressions of TGF‐β1/ Smad‐2/3*

The protein expression of TGF β in L^4^ centrum was measured. Conventional paraffin slice preparation and drying were conducted on L^4^ centrums. The slices were put into pre‐configured PBS solution and were washed twice for 5 min. Then the slices were treated with BSA/PBS sealing solution and incubated at room temperature for 30 min. Next, the slices were placed in a wet box and incubated at room temperature for 1 h with anti‐TGF β1 antibody (model no. ab92486, dilution rate 1:100, Abcam, Shanghai, China). In addition, these slices were combined with polyclonal anti‐rabbit IgG antibody (dilution rate 1:1000), which was marked with Alexa Fluor 488 as the second antibody. Then, finally, glycerin was added to the slices for sealing and they were kept in a dark place, covered in tin foil. The expression of TGF β was observed under a Nikon C2 laser scanning confocal microscope. For fluorescence microscope observation, indirect immunofluorescence intensity was assessed using the semi‐quantitative method (negative = 0; weak positive = 2; moderate positive = 4; strong positive = 6).

With soft tissue and vertebra appendix being removed, L^5^ was selected to extract the total protein using the cell lysis method. The detected value of electrophoretic protein was analyzed using Scion Image software. With glyceraldehyde 3‐phosphate dehydrogenase (GAPDH) as the internal reference protein, the expression level of proteins was calculated using the gray‐scale value of the target protein**/**the gray‐scale value of GAPDH.

### *Detection on mRNA Expression of TGF‐β1/Smad‐2/3*

L^6^ was selected to grind with liquid nitrogen and we extracted total RNA using Trizol. We used a thermo full wavelength enzyme standard instrument for detecting D value of the protein solution, which was conducted by Coomassie Brilliant Blue G250 (CBB G250) and adjusted the protein concentration of samples. The following operational processes were SDS‐PAGE gel electrophoresis, PVDF membrane transfer printing, incubation with primary antibody and second antibody, and enzyme color development. Imaging was done using an automatic gel imaging system (Tanon‐2500R), gray‐scale vRNA concentration and purity, and a Prime Script II 1st Strand cDNA Synthesis Kit for polymerase chain reaction (PCR) (Agilent Stratagene, model NO. Mx3000P) according to the operation steps of SYBR PremixEx Taq TM II. Reaction condition was set to 95°C for 30 s, 95°C for 5 s, and 60°C for 1 m, repeated 40 times, respectively. Every sample had three tec cDNA synthesis reactions. A quantitative reverse transcription‐PCR experiment was carried out. The primer sequences for amplification are shown in Table 1. The primers of TGF‐β1, Smad‐2/3 were supplied by America Life Corporation (Shanghai, China).

**Table 1:** Sequences of primers used in the real‐time polymerase chain reaction

Genes	Forward primer sequence (5′–3′)	Reverse primer sequence (5′–3′)
TGF‐β1	GCCTTCTCCATGGTGGTGAA	GGTCGGTGTGAACGGATTTG
Smad‐2	CCAGGTCTCTTGATGGTCGT	CTTGCTGTACTGTGTGTCCAGGC
Smad‐3	CAGGGCTTTGAGGCTGTCTA	GGTGCTGGTCACTGTCTGTC
GAPDH	GCCTTCTCCATGGTGGTGAA	GGTCGGTGTGAACGGATTTG

Note: GAPDH, glyceraldehyde 3‐phosphate dehydrogenase; TGF‐β1, transforming growth factor‐β1.

### *Statistics Analysis*

All statistical analyses were performed using SPSS software (version 22.0; IBM). A χ^2^‐test was used to identify differences between categorical variables and outcomes. Continuous variables were reported as means with standard deviations or medians with interquartile ranges. To compare data between groups, separate independent Student *t*‐tests were conducted for dependent variables that were normally distributed, and separate Mann–Whitney *U*‐tests were conducted for variables that were not normally distributed (α = 0.05). For all tests, *P* < 0.05 indicated statistical significance.

## Results

### *Histopathological Analysis*

**Figure 1:**
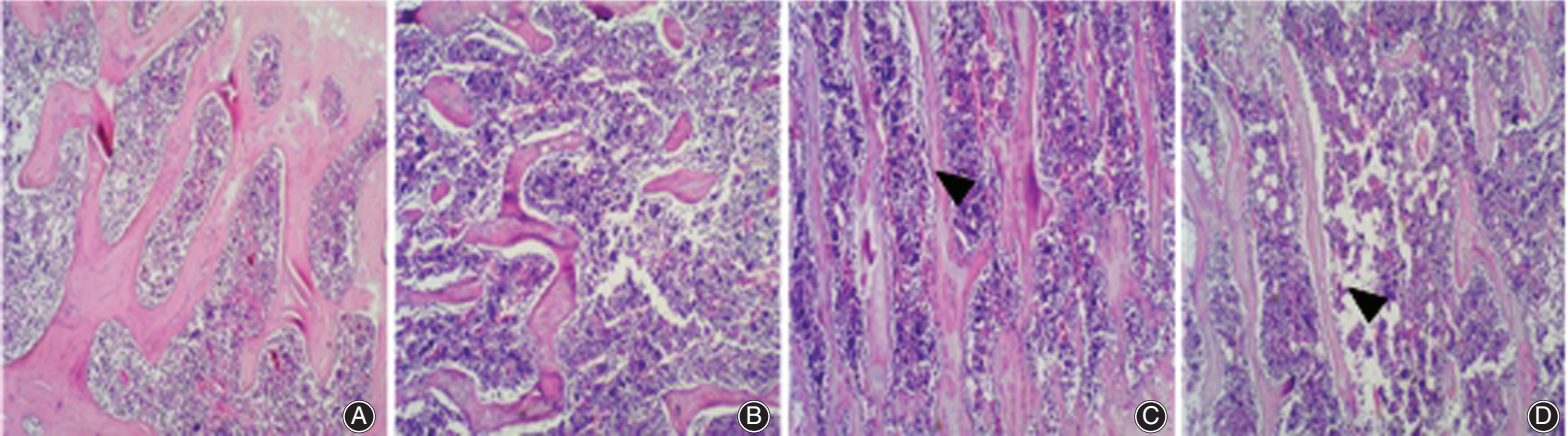
Comparison of hematoxylin and eosin (HE) results on femoral bone tissue: (A) group A, (B) group B, (C) group C and (D) group D. HE staining indicated that the morphological structures of the trabeculae in the left distal femora were improved compared with those in group B, and showed complete trabecular structure, decreased empty bone lacunae, fewer fractures, and slightly ordered trabeculae.

Hematoxylin and eosin staining indicated that in group B, the left distal femurs had sparse trabeculain uneven thickness, incomplete trabecular structure, disordered trabecular arrangement, and the remarkably increase bone lacunae compared with group A. After 12 weeks of treatment with AAT with TGP and Western medicine, the morphological structures of the trabeculae in the left distal femora were improved compared with those in group B, and showed complete trabecular structure, decreased empty bone lacunae, fewer fractures, and slightly ordered trabeculae (Fig. 1).

### *Electron Microscope Scanning on Bone Tissue*

**Figure 2:**
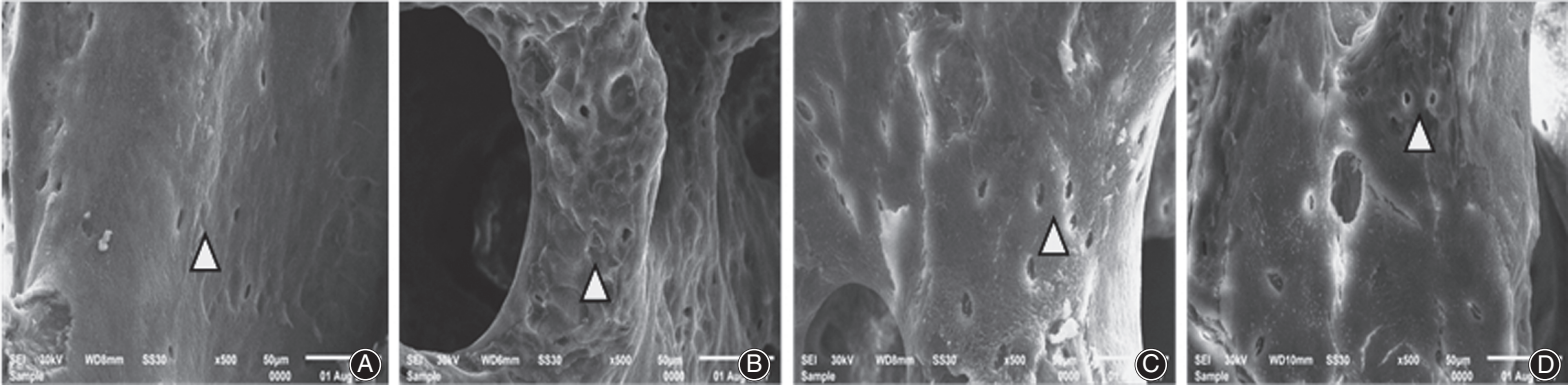
Comparison of electron microscope scanning results on femoral bone tissue (×30): (A) group A, (B) group B, (C) group C and (D) group D. Compared with group B, there was neatly arranged bone trabecula with slightly smaller bone resorption holes in group C, and neatly arranged thinner bone trabecula in group D.

**Figure 3:**
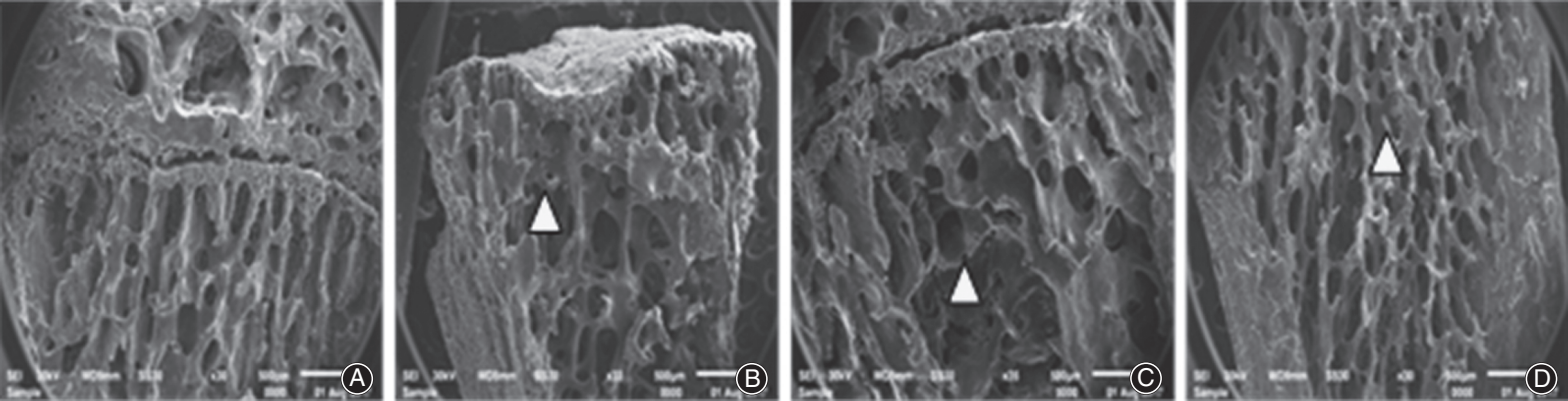
Comparison of electron microscope scanning results on femoral bone tissue (×500): (A) group A, (B) group B, (C) group C and (D) group D. The surfaces were relatively smooth in group C and D compared with group B.

**Figure 4:**
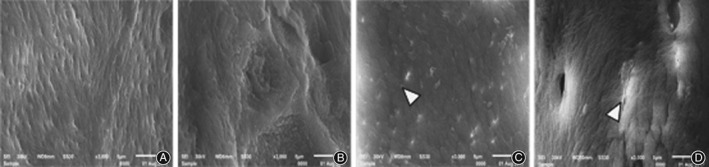
Comparison of electron microscope scanning results on femoral bone tissue (×3000): (A) group A, (B) group B, (C) group C and (D) group D. The arrangement of collagen fiber was relatively neat without apparent pits in group C and was relatively neat with shallow pits in group D.

Electron microscope scanning indicated that in group B, the right distal femurs showed uneven thickness with increased space, and the bone resorption holes were significantly enlarged. Compared with group B, there was neatly arranged bone trabecula with slightly smaller bone resorption holes in group C, and neatly arranged thinner bone trabecula in group D (Fig. 2). At high magnification (×500), the surfaces were relatively smooth in group C and D compared with group B (Fig. 3). In addition, at superfine magnification (×3000), the arrangement of collagen fiber was relatively neat without apparent pits in group C and was relatively neat with shallow pits in group D (Fig. 4).

### *Bone mineral density and Serum Bone Metabolism Markers*

In our study, we found a clear decrease in local BMD in groups B, C and D (respectively, 0.324 ± 0.049, 0.363 ± 0.045, and 0.356 ± 0.038 g/cm^2^). Compared with group B, there was a significant difference in both group C and D (*P* < 0.05).

As for bone metabolism markers, compared with group A, the level of BGP, BALP, and TRAP‐5b increased in groups B, C, and D (*P* < 0.05). Moreover, compared with group B, there was an obvious difference in the level of BGP, BALP and TRAP‐5b in both group C (respectively, 107.3 ± 7.6 pg/mL, 2.47 ± 0.39 ng/mL, and 0.355 ± 0.023 ng/mL) and group D (respectively, 113.0 ± 8.0 pg/mL, 2.74 ± 0.34 ng/mL and 0.361 ± 0.031 ng/mL) (Table 2).

**Table 2:** The results of bone mineral density and serum BALP, BGP, TRAP‐5b (mean±SD)

Groups	BMD (g/cm^2^)	BALP (ng/mL)	BGP (pg/mL)	TRAP‐5b (ng/mL)
Group A	0.409 ± 0.048	1.94 ± 0.20	105.4 ± 6.4	0.353 ± 0.032
Group B	0.324 ± 0.049▽▽	3.02 ± 0.41▽▽	119.2 ± 8.2▽▽	0.381 ± 0.025▽▽
Group C	0.363 ± 0.045※	2.47 ± 0.39※※	107.3 ± 7.6※※	0.355 ± 0.023※※
Group D	0.356 ± 0.038※	2.74 ± 0.34※	113.0 ± 8.0※	0.361 ± 0.031※

Note: Compared with the normal group (Group A),

▽▽ *P* < 0.01; compared with the model group (Group B),

※ *P* < 0.05,

※※ *P* < 0.01.

### *Protein Expression Assessment in Centrums*

**Figure 5:**
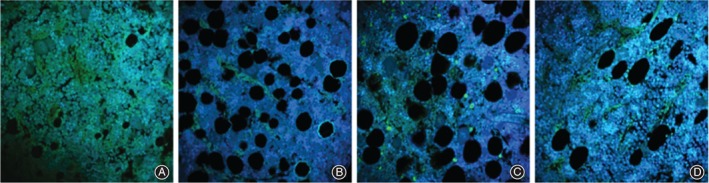
Comparison of TGF‐β1 in lumbar vertebra by immunofluorescent test: (A) group A, (B) group B, (C) group C and (D) group D.

**Figure 6:**
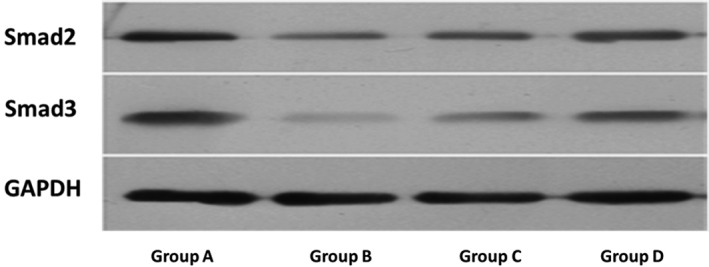
Comparison of the protein expressions of Smad‐2/3 in centrum.

**Figure 7:**
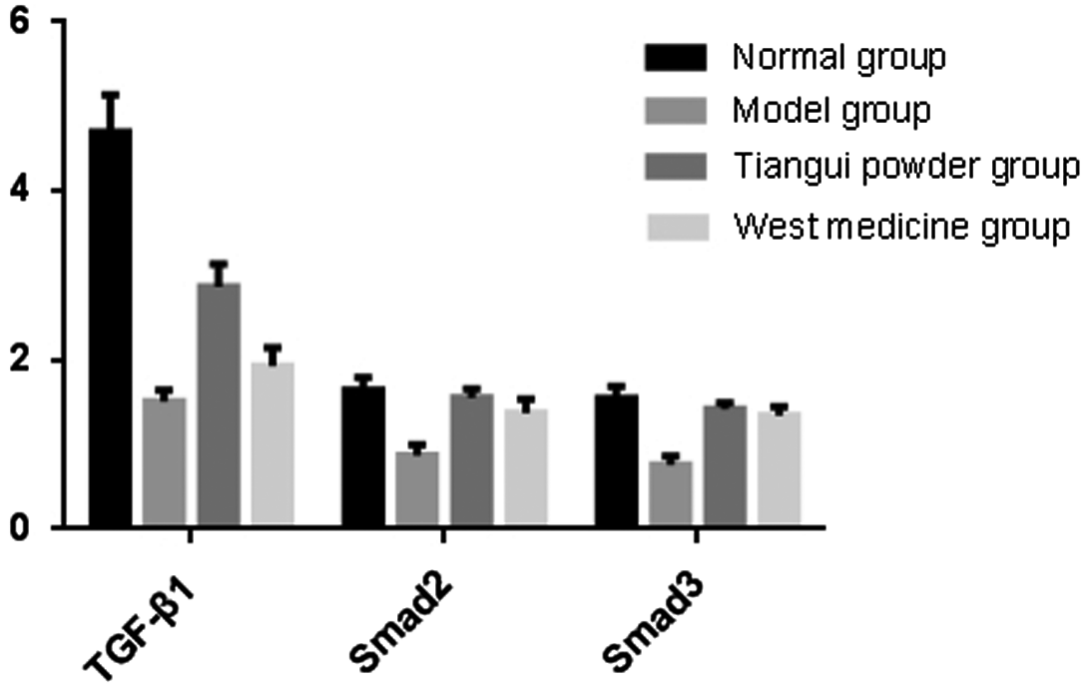
The protein expressions of TGF‐β1 and Smad‐2/3 in bone tissues.

As shown in Table 3, compared with group A, the level of protein expressions in TGF‐β1, Smad‐2/3 decreased in groups B, C, and D (*P* < 0.05). Compared with group B, the level of protein expressions in TGF‐β1, Smad‐2/3 were significantly increased in both group C (respectively, 2.870 ± 0.270, 1.552 ± 0.111, and 1.420 ± 0.079) and D (respectively, 1.930 ± 0.220, 1.370 ± 0.169, and 1.342 ± 0.103) (*P* < 0.01) (Figs 5, 6, 7, Table 3).

**Table 3:** The protein expressions of TGF‐β1/Smad‐2/3 in bone tissues (mean±SD)

Groups	TGF‐β1	Smad‐2	Smad‐3
Group A	4.700 ± 0.440	1.648 ± 0.145	1.550 ± 0.142
Group B	1.510 ± 0.140**	0.874 ± 0.124**	0.760 ± 0.105**
Group C	2.870 ± 0.270▲▲	1.552 ± 0.111▲▲	1.420 ± 0.079▲▲
Group D	1.930 ± 0.220▲▲	1.370 ± 0.169▲▲	1.342 ± 0.103▲▲

Note: Compared with the normal group (Group A),

** *P* < 0.01; Compared with the model group (Group B),

▲▲ *P* < 0.01.

### *mRNA Expression in Centrums*

**Figure 8:**
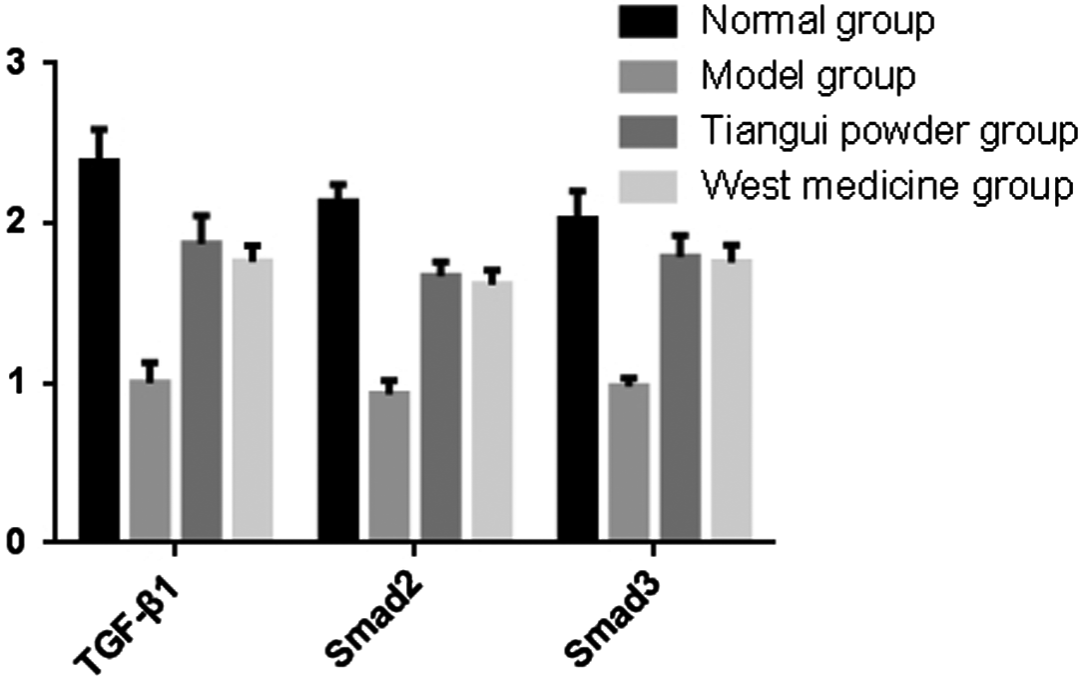
The mRNA expressions of TGF‐β1/Smad‐2/3 in bone tissues.

As the result of the mRNA expression in centrums showed, compared with group A, the mRNA expressions of TGF‐β1, Smad‐2/3 significantly decreased in group B, C, and D (*P* < 0.05). In addition, compared with group B, the difference of the mRNA expressions of TGF‐β1, Smad‐2/3 were significantly increased in both groups C (respectively, 1.872 ± 0.177, 1.672 ± 0.086, and 1.790 ± 0.136) and D (respectively, 1.762 ± 0.098, 1.616 ± 0.093, and 1.756 ± 0.108) (*P* < 0.01) (Fig. 8, Table 4).

**Table 4:** The mRNA expressions of TGF‐β1 and Smad‐2/3 in bone tissues (mean ± SD)

Groups	TGF‐β1	Smad‐2	Smad‐3
Group A	2.386 ± 0.202	2.136 ± 0.108	2.026 ± 0.179
Group B	1.002 ± 0.130**	0.932 ± 0.085**	0.978 ± 0.056**
Group C	1.872 ± 0.177▲▲	1.672 ± 0.086▲▲	1.790 ± 0.136▲▲
Group D	1.762 ± 0.098▲▲	1.616 ± 0.093▲▲	1.756 ± 0.108▲▲

Note: Compared with the normal group (Group A),

** *P* < 0.01; compared with the model group (Group B),

▲▲ *P* < 0.01.

## Discussion

### *Effect of Tonifying Kidney on Bone Formation*

According to the theory of TCM, PMOP is a chronic degenerative disease of the whole skeleton caused by deficiency of kidney essence and osteodystrophy^16^. Studies have shown that the method of tonifying the kidney can not only improve the clinical symptoms and inhibit bone absorption but also increase bone formation, regulate bone metabolism, and maintain the dynamic balance of bone formation and bone absorption by activating the TGF‐β1 and Smad signaling pathway^17^. Modern studies have also confirmed that PMOP is closely related to the decline of hormone levels in the hypothalamic–pituitary‐gonadal axis regulating system^18^. Many studies have indicated that AAT can regulate bone metabolism endocrine, improve bone metabolism, and increase bone density^11^, ^12^, ^13^, ^14^. This study shows that AAT with TGP and calcium supplement can effectively inhibit the decline of BMD and increase TGF‐β1, Smad‐2/3 expressions, improving morphology of bone tissue and bone metabolism in ovariectomized rats.

We have conducted many clinical studies on TGP in our hospital, and the results show that TGP could rebuild bone mass in postmenopausal women and obtain satisfactory outcomes. The medicines in this prescription nourish the kidneys, enrich marrow, and strengthen bones and muscles. Recent research has also suggested that the main Chinese medicines of the formula can not only inhibit bone absorption but also promote bone formation and slow down bone loss in PMOP alone or in combination^19^, ^20^, ^21^, ^22^. Moreover, Chinese medicines for nourishing kidneys and enriching marrow can increase BMD of PMOP model rats and prevent bone loss, which is demonstrated by this study.

### *Bone Morphology Changes*

Tissue morphology not only verifies the results of bone mass detection but also shows the conditions of osteoblasts, osteoclasts, bone cells, and marrow cavities in a more intuitive way^22^, ^23^. The results of this study showed that the microstructure and morphology of bone trabecula in model rats were damaged to different degrees, which is consistent with previous reports^24^, ^25^. The results showed that microstructure and bone tissue morphology were significantly improved through AAT with TGP, confirming the effectiveness of AAT with TGP and provided experimental evidence for its clinical application in PMOP.

### *Effect on Bone Metabolism Markers and TGF‐β1/Smad‐2/3 Signaling Pathway*

The change in bone transformation markers represents the dynamic state of bone metabolism^26^. It has been reported that BALP (marker of bone formation) and TRAP‐5b (marker of bone formation and absorption) can better reflect the changes of bone metabolism^27^, ^28^, ^29^, ^30^. As an important factor in pathophysiological changes, bone turnover level increased significantly in PMOP, the type I primary osteoporosis^3^. This study found that levels of serum BALP, TRAP‐5b, and BGP were significantly increased in the model rats, indicating an increase in bone turnover level that is in accordance with previous research. In addition, the AAT with TGP can significantly reduce the bone turnover rate and, thus, prevent bone loss.

The TGF‐β1 and Smad signaling pathway plays an important role in osteogenesis differentiation and bone homeostasis^31^, ^32^. Therefore, the present study was carried out by evaluating the mRNA and protein expression levels of TGF‐β1, Smad‐2/3 to further explore the pathogenesis of PMOP and the action mechanism of TGP. The mechanism of TGF‐β1 is as follows: the ligands combine with TGF‐β receptors in cytomembrane, and type I receptors are activated; Type I receptors transmit signals to the intracellular media Smad‐2/3; then those phosphorylated receptors of Smad‐2/3 combine with Smad‐4 and transduce TGF‐β1 signals directly from the cytomembrane to the cell nucleus to control the expression of downstream target genes. Smad‐2/3, the specific key factors in the TGF‐β signal transduction pathway^33^, play a significant role in formation, remodeling, and maintenance of bone^34^, ^35^, ^36^. In this study, the expressions of TGF‐β1, Smad‐2/3 in group B were significantly increased compared with group A, suggesting that the mechanism of PMOP was related to the abnormal expression of transduction factors in the TGF‐β1 and Smad signaling pathway. After the intervention of TGP, the expressions of TGF‐β1, Smad‐2/3 were significantly higher than that of group B, implying that the TGF‐β1 and Smad signaling pathway could be an important action target for TGP to treat PMOP. However, further attention should be paid to the mechanism of TGP in the downstream of TGF‐β1/Smad‐2/3 signaling pathway, especially the effect of TGP in osteogenic factors.

In summary, AAT with TGP could significantly improve the BMD of rats and repair the damaged bone microstructure and morphological structure in bone tissue by means of reducing the high turnover rate and prevent the bone loss of PMOP. Moreover, the TGF‐β1 and Smad‐2/3 signaling pathway could be a therapeutic target of TGP for PMOP.
